# Exophytic Mass Around the Tip of the Nose: Unusual Presentation of Metastatic Renal Cell Carcinoma

**DOI:** 10.7759/cureus.23113

**Published:** 2022-03-13

**Authors:** GS Randhawa, Dharmesh JB, Khushdeep Kaur

**Affiliations:** 1 General Surgery, Maharishi Markandeshwar Institute of Medical Sciences and Research, Mullana, IND; 2 Medicince, Maharishi Markandeshwar Institute of Medical Sciences and Research, Mullana, IND

**Keywords:** extra nasal mass, nasal neoplasms, exophytic, late metastasis, papillary rcc, renal cell carcinoma (rcc)

## Abstract

Renal cell carcinoma (RCC) has a predilection for metastatic spread, aggressive behavior. RCC is known for recurrence even after years of presentation. RCC has been linked to uncommon metastatic locations and unusual presenting symptoms from the disseminated illness. Common sites of metastasis include the lungs, liver, bones, brain, and adrenal glands, and many case reports are explaining the ability of RCCs to occur almost anywhere in the body. This case report states the unusual site of recurrence of RCC metastatic spread around the tip of the nose as exophytic growth years following nephrectomy. No such case is reported in the literature. We submit this case to report its occurrence, emphasize the rarity, presentation, diagnostic and therapeutic challenges, as well as a review of the literature. Renal cell carcinoma is the third most common cause of distant head and neck metastases and should be considered in the differential diagnosis of rapidly growing head and neck lesions.

## Introduction

Renal cell carcinoma (RCC) can have a wide range of clinical symptoms and might be difficult to diagnose because of its atypical presentation. The same is true for tumor recurrence, even years after the main tumor has been removed. Estimated new cancer cases of the kidney and renal pelvis account for 5% of male malignancies and 3% of female malignancies [1]. Tumors that originate in the kidney are present with various types of histological patterns, benign or malignant. As the use of imaging techniques has widespread applications, it has led to an increased diagnosis of renal cell carcinoma. Late metastases, years after the treatment of primary tumors, remain a well-known fact [2]. At the time of diagnosis, around 16% of RCC patients had metastatic disease [3]. Accordingly, metastatic RCC is referred to as a "clinical chameleon" since it can spread to practically every organ in the body, including the thyroid, pancreas, spleen, skin, gut, heart, and urinary bladder [4]. Even years after the original diagnosis, metastatic illness in these organs frequently manifests as metachronous metastasis [5]. Cancer cells leave the main tumor through the blood or lymphatics and are deposited at distant locations, resulting in the development of metastatic illness. This metastasis route is unpredictable, especially in renal carcinoma. The present case report depicts the most unusual site of recurrence of RCC on the tip of the nose as exophytic growth. No such case is reported in the literature.

## Case presentation

**Figure 1:**
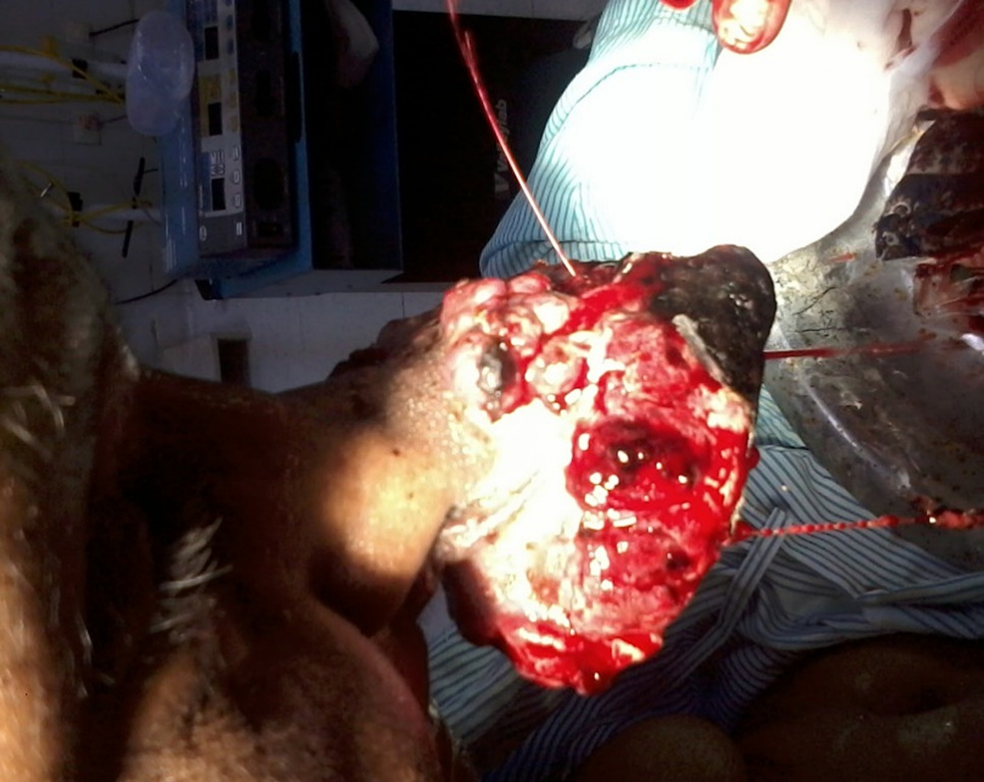
Preoperative picture

A 50-year-old female presented with an exophytic mass around the tip of the nose for the last three months (see Figure 1). The mass bled on touch.

**Figure 2:**
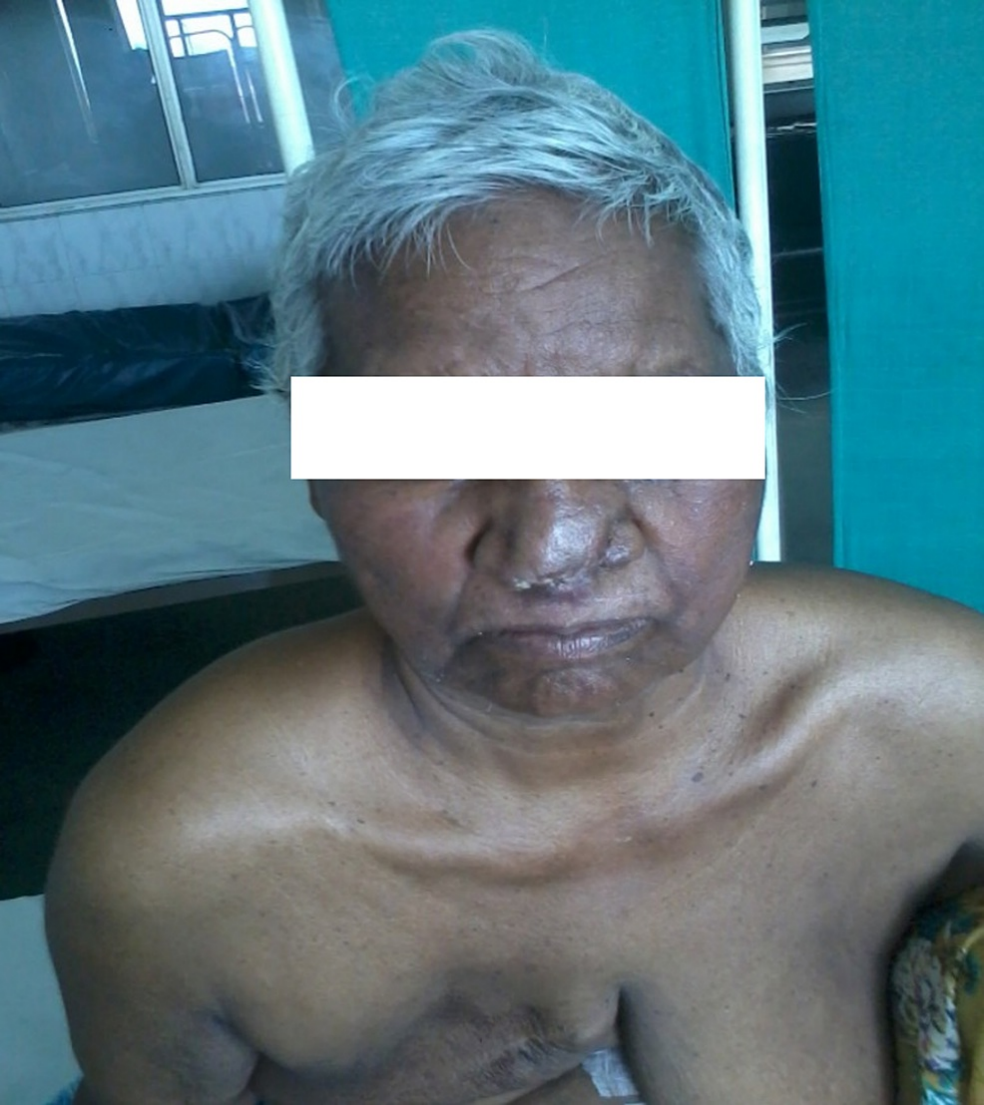
Postoperative picture

There was no history of nasal obstruction, anosmia or hyposmia, rhinorrhea, pain in and around the nose, and headache. The patient was a known case of invasive ductal carcinoma of the right breast diagnosed two years back, and she underwent right modified radical mastectomy followed by chemotherapy and hormonal therapy. The patient was also a known case of renal cell carcinoma for which she underwent partial nephrectomy 15 years back in another hospital, but she did not give a history of chemotherapy or radiotherapy for renal cell carcinoma. Her vitals were pulse rate 90/min, blood pressure 150/90mmhg, respiratory rate 20/min. Pallor was present. Local examination findings were an exophytic mass 9.5cm x 8.8cm of size around the tip of the nose, irregular shape and surface, ill-defined borders, active bleeding from the surface. Anterior and posterior rhinoscopy was performed, and no mass was seen in the nasal cavity. There was no redness, swelling, mass, or sinus in the area of paranasal sinuses. Ears, pharynx, and neck examination was normal. No regional lymphadenopathy was noted. Blood investigations revealed Hb 4 gm%, renal and hepatic functions tests were normal. Contrast-enhanced computed tomography (CECT) of paranasal sinuses showed no involvement of any paranasal sinuses or intranasal growth. Ultrasound of abdomen was normal. Chest X-ray was normal. The patient received multiple blood transfusions. Excision of the tumor was done under local anesthesia. The wound was closed primarily. The postoperative period was uneventful (Figure 2). Sutures were removed on the seventh postoperative day. Histopathological examination revealed papillary renal cell carcinoma. Her general health improved. The cosmetic appearance was not disturbed. The patient was shifted to the oncology ward for adjuvant chemotherapy. 

## Discussion

Renal cell carcinoma (RCC) is the most common malignancy in adults, accounting for 3% of all malignancies and 85% of all kidney tumors. The incidence of RCC is lower in Asia, especially in India, owing to a lack of reporting. Most RCC data comes from Western nations, and data from India, particularly about para-neoplastic disorders, is limited [6]. RCC recurrence after a long period is not uncommon. Patients with late recurrence exhibited superior clinicopathological characteristics and a better prognosis, with lengthy cancer-specific survival following recurrence [2].

Patients with distant metastases are prevalent, with 16% presenting with distant metastatic illness [3]. In less than 10% of patients, the triad of renal cell carcinoma (flank pain, gross hematuria, and palpable abdominal mass) is seen [7]. RCC metastasis to the head and neck region constitutes 6% [8]. In 7.5% of RCC patients, head and neck metastases were the primary complaint [9]. Papillary renal cell carcinoma (PRCC) is the second most frequent type of renal cell carcinoma (RCC) after clear cell RCC. It accounts for 6-18% of all RCC cases [10].

Tumors have a very intricate system of interconnected events that helps them maintain a growing cell mass and survive at the metastasis location. Tumor cells have a metastatic proclivity towards particular organs where the stromal environment is conducive to colonization. Effective colonization relies on tumor cell adaptability to the new environment by altering its epigenetic landscape. Solid-organ tumors metastasize sequentially [11]. Metastases occur from primary tumor to distant site via lymphatics and bloodstream. Most of these solid organ tumors metastasize to known distant sites/organs peculiar to their nature, but RCC is known for unusual sites.

After lung cancer and breast cancer, renal cell carcinoma (RCC) is the third most frequent metastatic tumor that spreads to the head and neck [12]. The initial tumor's clinical history is frequently unexpected, with spontaneous regression observed. Metastases may be discovered at the time of diagnosis in 25-30% of patients or at a later time following nephrectomy. Late metastasis after 17 years after nephrectomy has been observed in the literature [13]. Very few cases are reported in the ocular region, parotid gland, tongue, tonsils, thyroid, heart, uterus, testis, and ovaries. There were about 50 cases of renal cell carcinoma with nasal metastases in the literature [14]. The maxillary sinus is the most prevalent location for metastases in the paranasal group, followed by the ethmoid, frontal, and sphenoid sinuses [14]. Tumor involvement of the nasal and paranasal sinuses occurs through the hematogenous route by Baston's paravertebral plexuses. Raised intra-abdominal and intra-thoracic pressure causes increased flow to the paravertebral venous plexus, from which to venous sinuses of calotte and retrogradely to the pterygoid venous plexus before the paranasal sinus. This hypothesis describes how tumor cells can get past the pulmonary capillary filter and how the renal, pulmonary, and genitourinary systems and breast cancer metastasize to paranasal sinuses [15]. One of the defining features of our patient was that she was treated for carcinoma breast for which she had undergone modified radical mastectomy and chemo-radiotherapy. Even breast cancer is known to spread to unusual sites; an initial differential diagnosis was breast cancer metastases to the nose, and after the histopathological reporting, final diagnoses were made.

Surgery as a treatment option for metastasized RCC is quite significant. When metastases emerge more than two years following treatment of the main tumor, and there is appropriate surgical access, good oncologic clearance is accomplished. Head and neck metastases should be considered differently because of issues related to disease and quality of life. In situations of airway obstruction, bleeding, or discomfort, surgical management of head and neck metastases may be indicated for symptom control. All patients who are fit for surgery for both curative and palliative purposes should consider surgical care of such metastases [16].

Some authors recommend selective embolization before tumor biopsy, especially if the patient has a history of nephrectomy [15]. Patients with metastatic RCC are often treated both surgically and medically. Typically, patients receive a nephrectomy before the commencement of systemic chemotherapy. Median survival is five to 30 months following diagnosis, depending on the degree of the patient's neoplastic illness [9]. Although radiotherapy and immunochemotherapy have been suggested as treatment modalities for metastatic diseases, surgery remains the mainstay of treatment because most metastatic tumors in the nasal or paranasal sinuses are single [17].

## Conclusions

RCC has a predilection for metastatic spread and aggressive behavior. Because the patterns of metastases from RCCs are still being established, RCC has been linked to uncommon metastatic locations and unusual presenting symptoms from the disseminated illness. This case report states a rare instance of RCC metastatic spread around the tip of the nose years following nephrectomy. The Batson venous plexus is an anatomical route through which an embolus can travel to the head and neck and avoid pulmonary vascular filtration. Renal cell carcinoma is the third most common cause of distant head and neck metastases and should be considered in the differential diagnosis of rapidly growing head and neck lesions. We believe that local resection is the method of choice. This provides an opportunity to improve quality of life while treating head and neck metastases and is justified based on comorbidities that can occur if the lesion is left untreated.

## References

[1] Siegel RL, Miller KD, Fuchs HE, Jemal A (2021). Cancer Statistics, 2021. CA Cancer J Clin.

[2] Park YH, Baik KD, Lee YJ, Ku JH, Kim HH, Kwak C (2012). Late recurrence of renal cell carcinoma >5 years after surgery: clinicopathological characteristics and prognosis. BJU Int.

[3] (2022). Surveillance, Epidemiology, and End Results Program - cancer stat facts: kidney and renal pelvis cancer. https://seer.cancer.gov/statfacts/html/kidrp.html.

[4] Choucair K, Parker NA, Al-Obaidi A, Alderson J, Truong P (2020). Solitary, late metastatic recurrence of renal cell carcinoma to the pancreas: a case report. Cureus.

[5] Babar M, Hamdani S, Liu C, Vedula J, Schnapp DS (2019). Metachronous renal cell carcinoma with metastasis to the urinary bladder, and distant organs, 28 years after radical nephrectomy: a case report. BMC Urol.

[6] Pallagani L, Choudhary GR, Himanshu P (2021). Epidemiology and clinicopathological profile of renal cell carcinoma: a review from tertiary care referral centre. J Kidney Cancer VHL.

[7] Kruck S, Scharpf M, Stenzl A, Bedke J (2013). A rare case of synchronous renal cell carcinoma of the bladder presenting with gross hematuria. Rare Tumors.

[8] Morvan JB, Veyrières JB, Mimouni O, Cathelinaud O, Allali L, Verdalle P (2011). Clear-cell renal carcinoma metastasis to the base of the tongue and sphenoid sinus: two very rare atypical ENT locations. Eur Ann Otorhinolaryngol Head Neck Dis.

[9] Lenkeit C, Bank J, Shirazi M (2020). Renal cell carcinoma in the head and neck: case presentation of a patient with a rare metastatic pattern. Cureus.

[10] Le X, Wang XB, Zhao H, Chen RF, Ge P (2020). Comparison of clinicopathologic parameters and oncologic outcomes between type 1 and type 2 papillary renal cell carcinoma. BMC Urol.

[11] Mortezaee K (2021). Organ tropism in solid tumor metastasis: an updated review. Future Oncol.

[12] Serra A, Caltabiano R, Giorlandino A (2017). Nasal metastasis as the first manifestation of a metachronous bilateral renal cell carcinoma. Pathologica.

[13] Ziari M, Shen S, Amato RJ, Teh BS (2006). Metastatic renal cell carcinoma to the nose and ethmoid sinus. Urology.

[14] Sountoulides P, Metaxa L, Cindolo L (2011). Atypical presentations and rare metastatic sites of renal cell carcinoma: a review of case reports. J Med Case Rep.

[15] Torres Muros B, Solano Romero JR, Baró Rodriguez JG, Bonilla Parrilla R (2006). Maxillary sinus metastasis of renal cell carcinoma. Actas Urol Esp.

[16] Lieder A, Guenzel T, Lebentrau S, Schneider C, Franzen A (2017). Diagnostic relevance of metastatic renal cell carcinoma in the head and neck: an evaluation of 22 cases in 671 patients. Int Braz J Urol.

[17] Mahajan R, Mayappa N, Prashanth V (2016). Metastatic renal cell carcinoma presenting as nasal mass: case report and review of literature. Indian J Otolaryngol Head Neck Surg.

